# On Modality Effects in Bilingual Emotional Language Processing: Evidence from Galvanic Skin Response

**DOI:** 10.1007/s10936-017-9552-5

**Published:** 2017-12-28

**Authors:** Katarzyna Jankowiak, Paweł Korpal

**Affiliations:** Faculty of English, Adam Mickiewicz University in Poznań, al. Niepodległości 4, 61-874 Poznan, Poland

**Keywords:** Bilingualism, Emotional language processing, Modality effects, Galvanic skin response

## Abstract

Though previous research has shown a decreased sensitivity to emotionally-laden linguistic stimuli presented in the non-native (L2) compared to the native language (L1), studies conducted thus far have not examined how different modalities influence bilingual emotional language processing. The present experiment was therefore aimed at investigating how late proficient Polish (L1)–English (L2) bilinguals process emotionally-laden narratives presented in L1 and L2, in the visual and auditory modality. To this aim, we employed the galvanic skin response (GSR) method and a self-report measure (Polish adaptation of the PANAS questionnaire). The GSR findings showed a reduced galvanic skin response to L2 relative to L1, thus suggesting a decreased reactivity to emotional stimuli in L2. Additionally, we observed a more pronounced skin conductance level to visual than auditory stimuli, yet only in L1, which might be accounted for by a self-reference effect that may have been modulated by both language and modality.

## Introduction

Previous studies into bilingual emotional language processing have repeatedly pointed to a decreased sensitivity to emotionally-laden stimuli presented in the non-native (L2) relative to the native (L1) tongue (Harris 2004; Caldwell-Harris and Ayçiçeği-Dinn 2009; Opitz and Degner 2012; Pavlenko 2012; Caldwell-Harris 2014; Costa et al. 2014; Chen et al. 2015; Hsu et al. 2015). Such findings were observed not only in how bilingual participants self-reported their emotional reactivity to L1 compared to L2 materials (Bond and Lai 1986; Dewaele 2004; Pavlenko 2005; Puntoni et al. 2009), but also in experimental studies (Gonzalez-Regiosa 1976; Anooshian and Hertel 1994; Ayçiçeği and Harris 2004; Eilola et al. 2007; Sutton et al. 2007).

Such a foreign language effect has been argued to be related with a psychological distance when processing L2 emotionally-laden stimuli (Costa et al. 2014), and has been postulated to result from a number of different factors, including L2 proficiency level, age of L2 acquisition (AoA), as well as the context of L2 acquisition (Harris 2004; Caldwell-Harris 2014; Costa et al. 2014). The role of L2 proficiency level has been supported in a study by Costa et al. (2014), who adopted the “trolley dilemma” (Thomson 1985) and found that when making decisions, bilingual speakers were more moral in L1, and more utilitarian in L2. Importantly, such a difference between L1 and L2 was significantly smaller in the group of more proficient bilingual speakers, therefore indicating that an increase in L2 proficiency level might promote emotional grounding, which may consequently lead to a similar emotional sensitivity to both the native and non-native tongue (Costa et al. 2014).

Other researchers have however postulated that not only high L2 proficiency, but also early AoA are required in order for bilinguals to have a comparable emotional responding in L1 and L2 (Harris et al. 2006). Namely, research has shown a similar skin conductance response to L1 and L2 taboo words and reprimands in early bilinguals, but not in sequential bilinguals, who have acquired their L2 later than their L1 (Harris 2004), thus indicating that the response of the autonomic nervous system to affective words might be modulated by AoA. This is in line with the assumption that early language development occurs at the same time as the development of emotional regulation systems (Bloom and Beckwith 1989), as a result of which early-acquired lexical items might become more tightly connected with the emotional system of the brain (Caldwell-Harris 2014).

More recently, Caldwell-Harris (2014) proposed the emotional context of learning hypothesis, which postulates that the context in which a language is learned is another factor that might modulate emotional resonance. Namely, a language is assumed to be perceived as more emotional when it has been acquired and is used in emotional contexts. The importance of context-dependent learning is consistent with studies showing that L2 becomes more emotional when it is learned via immersion rather than in the formal school setting (Dewaele 2010; Degner et al. 2012).

Importantly, additional support for the assumption of a decreased emotional reactivity when processing emotionally-laden materials in the foreign language has been provided by research that has employed the skin conductance method, showing a reduced galvanic skin response (GSR) to the non-native compared to the native tongue (Harris 2004; Caldwell-Harris and Ayçiçeği-Dinn 2009). GSR is an autonomic measure of emotion based on electrodermal responses, and follows the assumption that sweating reflected in an increased skin conductance is a marker of physiological arousal, indexing the activity of the autonomous nervous system (ANS). For this reason, GSR is frequently employed as a marker of emotional arousal (Bradley et al. 1990; Cook et al. 1991; Waugh et al. 2011; Monfort et al. 2014), and is often used to measure the physiological arousal in response to experiencing basic emotions (i.e., anger, disgust, fear, happiness, sadness, surprise; Ekman 1999). Although basic emotions might vary on the arousal scale, with anger and fear usually described as high arousing emotions, and sadness as a low arousing emotion (e.g., Kensinger and Corkin 2004; Kensinger 2004), previous studies have shown that this difference might not necessarily be reflected in GSR patterns (e.g., Levenson et al. 1990; Kreibig et al. 2007; Vidmar et al. 2016), thus indicating that GSR signal might not be a clear indicator of which particular basic emotion is currently experienced.

Importantly, the activity of the ANS may not necessarily reflect emotional responding, but it might also index other functions of the organism, for example those related to attention and cognitive engagement (Frith and Allen 1983; Berntson and Cacioppo 2000; Mauss and Robinson 2009). As a result of such potential confounds, the GSR measurement is often combined with other measures of emotion processing, including self-reports, which instead of examining physiological arousal, allow for measuring cognitive labels (Schachter and Singer 1962; Waugh et al. 2011; Monfort et al. 2014).

Little attention has however been devoted to comparing how different modalities could modulate emotional language processing. Research on the role of modality in monolingual language processing has shown that visual and auditory linguistic stimuli may not be processed alike. For instance, Holcomb et al. (1992) examined event-related potentials (ERPs) elicited in response to sentences that were presented visually or auditorily, and which ended with either a highly expected or a semantically anomalous word. The findings showed more pronounced N400 amplitudes for anomalous items presented visually than auditorily, thus possibly indicating that visual stimuli might engage a greater activation in the semantic memory network relative to auditory stimuli. Furthermore, some studies have suggested that lexico-semantic mechanisms engaged in the processing of auditory stimuli might start earlier compared to visual materials, as reflected in the earlier onset of the N400 response to auditory relative to visual linguistic stimuli (Holcomb and Neville 1990; Lartseva et al. 2015). Potential differences in how visual and auditory linguistic stimuli are processed have also been observed in a functional magnetic resonance imaging (fMRI) study conducted by Buchweitz et al. (2009). The authors compared brain activation patterns when participants were either reading or listening to sentences, and observed a left-lateralized activation when participants were reading sentences as well as a more bilateral activation when they were listening to sentences.

In bilingual language processing, the effect of modality was investigated by Woutersen et al. (1995), who tested English–Dutch bilingual speakers in two lexical decision tasks using cognate words (words with a similar form and meaning in two languages) as well as non-cognate lexical items. While in one task, the authors employed an auditory repetition priming paradigm with stimuli presented via headphones, the other task involved a visual repetition priming paradigm, in which the stimuli were displayed on the computer screen. Surprisingly, no differences in interlingual priming between the two modalities were observed, which led the authors to reject the proposed “modality hypothesis” and conclude that “no different processing strategies are used for phonology and orthography” (Woutersen et al. 1995: 297). Such findings seem to be therefore contradictory to what previous research into the role of modality in monolingual language processing has shown.

To date, the role of modality in bilingual emotional language processing has been only little researched. In their seminal study, Harris et al. (2003) tested late proficient Turkish (L1)–English (L2) bilingual speakers, and recorded their skin conductance responses elicited to emotionally-laden items that were presented in their native and non-native tongue. In line with previous research, the authors observed more pronounced skin conductance responses (SCRs) for L1 relative to L2 materials. Interestingly, the effect of modality was observed, yet only in L1, in which emotionally-laden items presented in the auditory modality evoked an increased SCR. In a subsequent GSR study, Harris (2004) also used emotionally-laden items in the visual and auditory modality, but tested early as well as late Spanish (L1)–English (L2) bilingual speakers. Interestingly, in the group of early bilinguals, the author found a modality effect in both the native and non-native language, with a more pronounced GSR to the auditory modality. Altogether, the preference for the auditory modality observed in the findings obtained from the two experiments might have resulted from the fact that spoken language is acquired before visual language during L1 acquisition. Consequently, spoken language might be better connected to brain systems responsible for emotion processing. This may nonetheless not be the case for the non-native language, if it has been acquired later in life. Importantly, the stimuli used in the studies by Harris et al. (2003) as well as Harris (2004) included either single words or short reprimands, and thus the role of modality in the bilingual processing in emotionally-laden narratives remains under-investigated.

Thus far only little attention has been devoted to examining the role of modality in bilingual processing of emotionally-laden narratives, as opposed to single words. Hence, the main aim of the current experiment was to examine whether late proficient bilingual speakers are less sensitive to emotionally-laden narratives presented in their non-native (English) compared to the native tongue (Polish), and whether such effects are modulated by modality. Namely, we wanted to investigate if there is a preference for auditory stimuli when processing emotional language in both L1 and L2. To this aim, we used emotionally-laden narratives, which were presented to our participants in the visual (a text displayed on the computer screen) as well as auditory modality (an audio recording presented via headphones), in their L1 and L2. In order to limit the effect of a potential confounding variable, sadness was selected as one basic emotion to be evoked in all texts. Although so far, much attention has been devoted to studying sadness, for instance, by means of using sad pictures (e.g., Ritz et al. 2000; Rainville et al. 2006), videos (e.g., Gross and Levenson 1997; Tsai et al. 2000), and musical excerpts (e.g., Krumhansl 1997; Etzel et al. 2006), little research has been conducted on the processing of sad narratives.

In our study, we employed two measures of emotion: an autonomic measure (galvanic skin response) to examine physiological arousal, and a self-report measure (SUPIN S30—the Polish adaptation of PANAS—*Positive and Negative Affect Schedule*; Watson et al. 1988) to examine how participants label their emotions. First of all, we expected an attenuated emotional response to L2 stimuli, as reflected in a smaller GSR as well as lower SUPIN scores. Such findings would be in line with previous research, and would point to a decreased sensitivity to emotional stimuli in the non-native language due to differences in the age as well as context of L1 and L2 acquisition (Harris 2004; Harris et al. 2006; Dewaele 2010; Degner et al. 2012; Caldwell-Harris 2014).

Secondly, we hypothesized a more pronounced galvanic skin response along with a more robust negative affect to auditory stimuli (as indicated by the SUPIN measurement) in the native tongue, yet not in the non-native language. Such findings would be in line with previous research on the role of modality in bilingual emotional language processing, which has shown a preference for the auditory modality in L1 when testing late bilingual speakers, and in both L1 and L2 when testing early bilinguals (Harris et al. 2003; Harris 2004). Importantly, since previous research has employed either single words or short reprimands, in our study, we wanted to verify whether the same effect would be observed in late Polish (L1)–English (L2) bilinguals processing emotionally-laden narratives.

## Methods

### Participants

The original sample included 30 participants. Two of them had to be excluded from the analyses due to technical issues during GSR signal acquisition. Additionally, one participant had to be removed from further analyses owing to excessive hand movements during GSR data collection. This resulted in a final sample of 27 native speakers of Polish (16 women, 11 men; $M_{age}=26.10$, *SD* $=$ 3.54), who were students of the Faculty of English at Adam Mickiewicz University in Poznań, Poland. Participants recruited for the present study had all passed Practical English Language Exam, which was equivalent to Cambridge Proficiency Examination administered by Cambridge University. Participants were late proficient learners of English as their second language ($M_{{ AgeofAcquisition}}=9.07$, ${ SD}=3.00$), which they learnt in the formal school setting in Poland. Their high proficiency level in English was tested by means of administering an on-line LexTALE test (Lemhöfer and Broersma 2012; available at www.lextale.com), whose mean result equaled $M=85.98$, ${ SD}=6.61$ (LexTALE scores higher than 80% indicate C1/C2 proficiency level according to the Common European Framework of Reference for Languages). Apart from English, participants had been learning other foreign languages as their L3 ($M_{{ AgeofAcquisition}}=17.44$, ${ SD}=4.36$), such as German, French, Spanish, Russian, and Portuguese. All of the participants had normal or corrected to normal vision, they did not suffer from any language, neurological, and psychiatric disorder, and they did not take drugs that would affect their nervous system.

### Materials

Materials used in the study consisted of 4 Polish and 4 English experimental texts, as well as 2 Polish and 2 English control texts. The experimental materials involved texts on topics such as death of a relative (Text 1), terminal illness (Text 2), physical abuse (Text 3), and loss of a child (Text 4). The control texts involved descriptions of cities and included demographic data, history of a city, as well as statistical and economic information. The stimuli included English–Polish translation equivalents, and were translated from English into Polish by two professional translators, who were native speakers of Polish. Importantly, Polish is a highly inflected language, and a suffix is added to every verb to indicate grammatical gender. As a result, in order to ensure consistency across all texts presented in the visual modality, they were always marked for female gender. As for the auditory materials, Polish texts were read and recorded by a female native speaker of Polish, while English texts were read and recorded by a female English native speaker. The criteria under which the texts were controlled for are provided in Table 1. Polish texts had a larger number of words compared to English text, which results from the fact that Polish is characterized by a more synthetic structure than English, which is reflected in the higher morpheme-per-word ratio. For example, due to a high degree of inflection in Polish, subject pronouns are frequently omitted, which is not the case in English.

**Table 1:** The characteristics of Polish and English texts used in the study

	Number of words	Mean sentence length	Text readability
Polish texts	226–243 $(M=237, { SD}=7.53)$	7–9 $(M=8, { SD}=1.15)$	1–2 $M=1.5, { SD}=.58$ (according to *Jasnopis,* where $1=\hbox {very easy}, 2=\hbox {easy}$)
English texts	243–251 $(M=247.5, { SD}=3.32)$	7.4–10.3 $(M=8.85, { SD}=1.37)$	90.4–97.7 $M=93.15, { SD}=3.43$ (according to the Flesch–Kincaid Index of readability, where texts above the index of 90.0 are considered to be easy)

### Normative Studies

Both Polish and English experimental texts in both the visual and auditory modality were pretested using web-based Likert-type surveys. Each survey was completed by 30 raters; yet, raters whose scores were more than 3 SDs from the mean were excluded from final analyses. While Polish experimental materials were evaluated by native speakers of Polish, English stimuli were assessed by both English native speakers and Polish learners of English as their second language. The raters who took part in these pretests did not participate in the experiment proper. Table 2 provides demographic information concerning raters whose ratings were included in the analyses.

**Table 2:** Demographic characteristics of raters of the normative studies

Text	Modality	Raters	Number of raters included in the analyses	Mean age	Age of L2 acquisition
Polish texts	Visual	Polish native speakers	$\hbox {N}=29$ (24 women, 5 men)	$M_{age}=22.69, { SD}=2.82$	*n* / *a*
	Auditory	Polish native speakers	$\hbox {N}=25$ (21 women, 4 men)	$M_{age}=22.2, { SD}=1.41$	*n* / *a*
English texts	Visual	English native speakers	$\hbox {N}=30$ (18 women, 12 men)	$M_{age}=23.40, { SD}=5.97$	*n* / *a*
	Auditory	English native speakers	$\hbox {N}=27$ (15 women, 12 men)	$M_{age}=23.29, { SD}=9.72$	*n* / *a*
English texts	Visual	Polish native speakers, L2 learners of English	$\hbox {N}=29$ (26 women, 3 men)	$M_{age}=21.96, { SD}=2.12$	$M_{age}=8.18, { SD}=2.12$
	Auditory	Polish native speakers, L2 learners of English	$\hbox {N}=27$ (24 women, 3 men)	$M_{age}=22.88, { SD}=1.77$	$M_{age}=8.69, { SD}=2.98$

With a view to ensuring that all of the experimental stimuli were perceived as evoking sadness, raters were asked to complete an online survey, in which they were supposed to rate the four experimental texts on a 5-point Likert scale ($1=\hbox {definitely non-sad}$, $5=\hbox {definitely sad}$). Visual and auditory stimuli were tested in separate surveys, and were evaluated by different respondents. The results showed that texts presented in the visual modality were perceived as sad in both Polish ($M=4.5$, ${ SE}=.01$) and English ($M=4.27$, ${ SE}=.01$), as reflected in no statistically significant differences between Polish and English texts, $t=1.6, { df}=3, p>.05$. Similar statistically significant results were obtained from the surveys on the auditory materials, which were also perceived as sad in both Polish ($M=4.66, { SE}=.11$) and English ($M=4.49, { SE}=.10$), $t=1.3, { df}=3, p>.05$. Differences were however observed between the stimuli presented in the auditory and visual modality, yet only in Polish, where the auditory texts were evaluated as more sad than the visual materials, $t=-\,5.43, { df}=3, p=.012$. No such differences were nonetheless found with regard to the English stimuli, $p>.05$.

Additionally, in order to verify whether L2 learners of English perceive the selected English stimuli as English native speakers do, another norming study was conducted, where Polish–English bilinguals (see Table 2) were asked to rate the English texts on a 5-point Likert scale ($1=\hbox {definitely}$ non-sad, $5=\hbox {definitely}$ sad). The findings revealed that English L2 learners also perceived the English texts as sad, in both the visual ($M=4.28, { SE}=.10$) and auditory modality ($M=4.73, { SE}=.10$), with however higher sadness ratings for the auditory relative to the visual modality, $t=-\,8.58, { df}=3,p=.003$. An independent samples *t* test confirmed that there was no statistically significant difference between the group of English native speakers and English L2 learners with regard to the stimuli presented in the visual modality, $p>.05$. As for the auditory modality, on the other hand, the findings showed that the materials were perceived as more sad for English L2 learners compared to English native speakers, $t=2.214, { df}=52, p=.031$.

To account for potential differences in how visual and auditory materials were perceived, a mixed-design analysis of variance (*ANOVA*) was performed with 4 experimental texts (Text 1 vs. Text 2 vs. Text 3 vs. Text 4) as a within-subject factor, as well as 2 modality (Visual vs. Auditory) and 3 group (Polish native speakers vs. English native speakers vs. English L2 learners) as between-subject factors. The findings showed an effect of modality, $F(1, 160)=11.05, p=.001, {\upeta }_{\mathrm{p}}^{2}=.065$, with higher sadness ratings for the auditory ($M=4.63, { SE}=.06$) relative to visual materials ($M=4.35, { SE}=.06$). The effects of group as well as the interaction between group and modality were statistically insignificant ($p>.05$). The results of the normative studies on the experimental stimuli are reported in Table 3.

**Table 3:** Results of the normative studies on the experimental stimuli

Text	Modality	Raters	Sadness
Polish texts	Visual	Polish native speakers	$M=4.5, { SE}=.01$
	Auditory	Polish native speakers	$M=4.66, { SE}=.11$
English texts	Visual	English native speakers	$M=4.27, { SE}=.01$
	Auditory	English native speakers	$M=4.49, { SE}=.10$
	Visual	Polish native speakers, L2 learners of English	$M=4.28, { SE}=.10$
	Auditory	Polish native speakers, L2 learners of English	$M=4.73, { SE}=.10$

### Procedure

The procedures applied in the experiment were in accordance with the ethical guidelines for research with human participants, and were approved by the Adam Mickiewicz University Human Research Ethics Committee. Participants were informed about the procedures of the experiment, and were asked to sign the informed consent form before the experiment began. They were also screened for potential language disorders, psychological disorders (e.g., depression), and visual impairments by means of filling in a self-report questionnaire. After the experiment, each participant was administered an on-line LexTALE test to ensure their high proficiency level in English as their second language (Lemhöfer and Broersma 2012).

Participants were seated in front of a computer screen and equipped with headphones. They were informed that their task would be to attentively read and listen to short narratives, which would be either displayed on the screen or played via headphones. Before the experiment, participants were not informed that the texts were aimed at evoking sadness in order not to elicit a priming effect. Participants were presented with 6 texts (4 experimental and 2 control). Each experiment consisted of 2 Polish and 2 English experimental texts (one presented visually and one auditorily in each language), whose presentation was pseudo-randomized, so that they could be grouped in two language blocks. The order of the presentation of the experimental stimuli (both their content and the modality in which they were provided) was counterbalanced across participants. Half the participants began the experiment with Polish items, while the other half were first presented with English stimuli. Two control texts (one visual and one auditory) were additionally presented as the final stimuli, in the language of the second block. The experiment ended with a 2-min resting state, during which participants were asked to look at a fixation cross displayed on the computer screen.

Visual stimuli were displayed using black letters (font size: 24, Times New Roman), and were presented on a gray background. Auditory stimuli were delivered through headphones with a pre-set volume. No visual stimuli were displayed on the computer screen when participants were listening to the auditory stimuli. While the presentation of the audio files lasted depending on their duration ($M=108\,\hbox {s}, { SD}=13.86$ for the Polish recordings, and $M=91\,\hbox {s}, { SD}=4.28$ for the English recordings), the presentation of the visual texts was self-paced. With a view to ensuring that participants processed presented stimuli at a semantic level, after each text (both a written text and an audio recording), participants were asked to answer two yes/no comprehension questions related to its content. As soon as they answered the questions related to a given text, participants were asked to fill in the SUPIN S30 questionnaire (Brzozowski 2010), which is the Polish adaptation of PANAS (*Positive and Negative Affect Schedule*; Watson et al. 1988), a self-report tool used to measure current emotional states. The questionnaire consists of 30 adjectives, out of which 15 represent negative emotions, and the remaining 15 represent positive emotions. Answers are given on a 5-point Likert scale, where “1” indicates that a given emotion is not experienced at all, and “5” indicates that the strength of the emotion experienced at a given point in time is very significant. Participants filled in the SUPIN S30 questionnaires after each experimental and control text. The experimental session took around 20–25 min.

### Galvanic Skin Response Recording

To measure skin conductance in the experiment, an ADInstruments GSR Amp galvanic skin response amplifier was used. Two 8 mm re-usable diameter electrodes were attached to medial phalanx of both the index and middle finger of the non-dominant hand of each participant. Participants were asked to try not to move their hand during the experiment.

GSR was measured for the whole duration of the experiment, that is, 4 experimental stimuli, 2 control texts, and the resting state. As participants processed visual stimuli at different speeds, we calculated a total response count per 1 min of each recording. Following recommended validity criteria for the software used, 0.02 $\upmu$S minimum limit for measured skin conductance was adopted (PsychLab 2003). Skin conductance was recorded by means of the PsychLab Data Acquisition software. Acquisition sample rate was set to 1000 Hz, and idle sample rate—500 Hz. The collected data were further analyzed using the PsychLab Analysis software. As required by the software, to calculate total response count, all the data were sectioned into blocks of 30 s each, and then processed to detect skin conductance responses in each moment of interest; namely, when the participants processed visual and auditory stimuli (4 experimental stimuli, 2 control texts, and the resting state), based on visual inspection.

## Results

### SUPIN S30

**Fig. 1:**
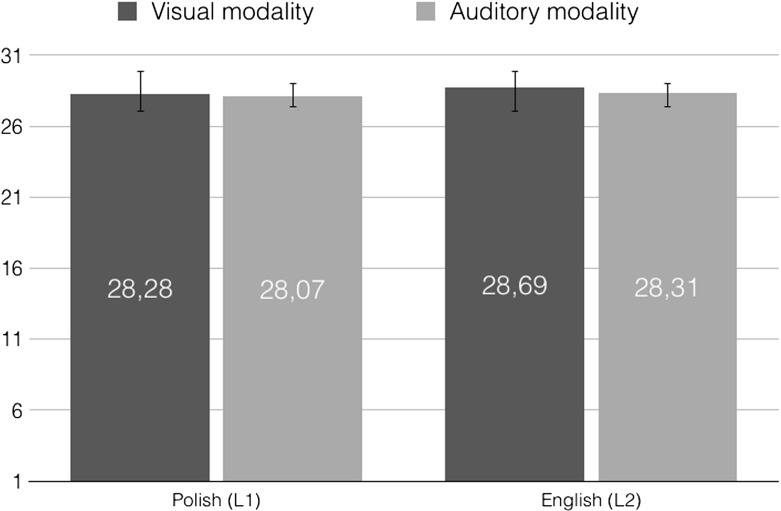
Mean SUPIN results for the visual (dark gray) and auditory (light gray) modality in Polish (left-hand side) and English (right-hand side)

The analysis performed on the self-ratings obtained from the SUPIN questionnaires was based on values for all negatively-valenced adjectives ($\hbox {N}=15$), such as *przygn* *ębiony* (Eng. sad), *zmartwiony* (Eng. upset). The Negative Affect Score was calculated in line with the scoring instructions (Brzozowski 2010). Once the scores were calculated, a 2 modality (visual vs. auditory) $\times$ language (Polish vs. English) repeated measures *ANOVA* was conducted, which however did not yield any statistically significant findings, $p>.05$. Mean SUPIN results per each modality in each language are provided in Fig. 1.

### Galvanic Skin Response

Before analyzing GSR responses to specific levels of an independent variable, we averaged the number of skin conductance responses to all experimental materials and compared them with the two control conditions (control texts and the resting state). An analysis of variance (*ANOVA*) with condition type as a factor showed a main effect of condition, $F(2, 52)=53.48, p<.001, {\upvarepsilon }=.67, {\upeta }_{\mathrm{p}}^{2}=.673$. Pairwise comparison further revealed that the experimental conditions ($M=6.08, { SE}=.67$) elicited a more pronounced skin conductance response compared to the control texts ($M=3.27, { SE}=.43$), $p<.001$, and the control texts evoked more robust GSRs than the resting state ($M=2.33, { SE}=.44$), $p=.008$. Additionally, in order to examine whether experimental conditions evoked more pronounced SCRs compared to control texts in both the native and non-native languages, as well as in both the visual and auditory modalities, paired-samples *t* tests were conducted. As expected, we observed a larger number of GSRs to experimental relative to control texts in L1 visual modality ($p=.001$), in L1 auditory modality ($p<.001$), in L2 visual modality ($p=.001$), and in L2 auditory modality ($p<.001$). Correction for multiple comparisons was applied here, and the critical *p* level for significance was set to .012. Finally, we did not observe any statistically significant differences between Polish and English control texts in both the visual ($p>.05$) and auditory modality ($p>.05$).

The number of skin conductance responses to the experimental texts were further analyzed by means of performing a 2 modality (visual vs. auditory) $\times$ language (Polish vs. English) repeated measures *ANOVA*. The analysis revealed an interaction between modality and language, $F(1, 26)=17.10, p<.001, {\upeta }_{\mathrm{p}}^{2}=.397$. Follow up paired samples *t* tests further showed that in Polish, texts presented in the visual modality ($M=7.70, { SE}=.91$) evoked an increased number of skin conductance responses relative to the auditory modality ($M=6.05, { SE}=.71$), $t=-\,3.29, p=.003, r=.294$. Such a difference was nonetheless not observed in English, where the visual ($M=5.10, { SE}=.66$) and auditory materials ($M=5.49, { SE}=.68$) elicited a similar number of skin conductance responses, $p>.05$. With regard to between-language differences, post-hoc tests revealed that the visual stimuli evoked a larger number of skin conductance responses in Polish (the native tongue) compared to English (the non-native tongue), $t=-\,5.99, p<.001, r=.579$. There was no statistically significant difference between Polish and English auditory materials, $p>.05$. Correction for multiple comparisons was applied here, as a result of which the critical *p* level for significance was set to .012.

**Fig. 2:**
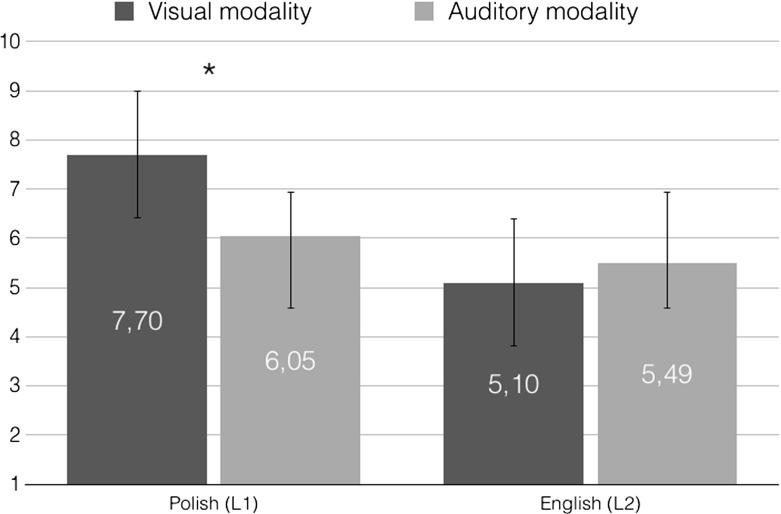
Mean number of galvanic skin responses to the visual (dark gray) and auditory (light gray) modality in Polish (left-hand side) and English (right-hand side)

In addition to the interaction, a main effect of language was found, $F(1, 26)=20.02, p<.001, {\upeta }_{\mathrm{p}}^{2}=.435$. Skin conductance responses were more pronounced in Polish ($M=6.87, { SE}=.78$) than in English ($M=5.29, { SE}=.65$). Mean number of skin conductance responses per each modality in each language is provided in Fig. 2.

## Discussion

The present experiment was aimed at elucidating the role of modality in emotional language processing in the native and non-native language. To this aim, we employed the galvanic skin response method along with a self-report measure (i.e., SUPIN S30) to investigate how highly proficient late bilingual speakers responded to emotionally-laden narratives presented in the visual and auditory modality, in their L1 and L2.

Contrary to what was expected, we found a more pronounced emotional response to the narratives presented in the visual modality, as indicated by an increased galvanic skin response; yet, this effect was observed only in the native language. Previous research into the role of modality in bilingual emotional language processing (Harris et al. 2003; Harris 2004) has pointed to a preference for auditory over visual emotional stimuli, either in L1 only (late bilinguals) or in both L1 and L2 (early bilinguals). On the contrary, the results of our study demonstrate a preference for the visual over auditory modality in the native language. Such divergent results might suggest that there is a different pattern of emotional response to narratives, as opposed to single words and short reprimands.

The preference for the visual over auditory modality in the processing of emotionally-laden narratives can be explained within the levels of processing theory (Craik and Lockhart 1972) and the self-reference effect (Rogers et al. 1977) that could potentially be modulated by both language and modality. The levels of processing theory (Craik and Lockhart 1972) is based on the assumption that information can be processed at a more shallow (structural, phonemic) and deep (semantic) level. The theory was developed to discuss information processing involved in memory and its main tenet is that the deeper the information is processed, the better it is remembered. In line with the self-reference effect (Rogers et al. 1977), information processing is even further facilitated when the information refers to ourselves, and this constitutes the deepest processing level. The self-reference effect can thus provide a plausible explanation for the visual dominance in the processing of emotional stimuli in L1, due to the fact that when reading the narratives, participants could refer the stories to their own experience, or imagine that the same series of unfortunate events could happen to them. On the contrary, when listening to an audio recording, participants were aware that the stories were told by an individual who had experienced the events. Even if they sympathized with the actor, they could refer the information to themselves to a more limited extent than when processing texts in the visual modality.

However, it needs to be noted that the findings observed in the experiment proper do not support the assessment of the stimuli in the normative tests, where auditory stimuli were evaluated as more sad than visual materials by Polish L1 speakers (texts in Polish), as well as English L2 learners (texts in English), and where no differences between the visual and auditory modality were observed (texts in English evaluated by English L1 speakers). This might be explained as resulting from the fact that the raters only evaluated the properties of the stimuli (i.e., the degree of sadness), and their emotional states were not measured. In the experiment proper, on the other hand, we investigated participants’ emotional reactivity to the presented texts, as measured by both physiological arousal and self-reported emotional states. Furthermore, a between-subject design was used in the normative tests, where raters were instructed to assess either visual or auditory materials, whereas participants of the experiment proper were all presented with stimuli in both modalities (a within-subject design), which thus might have provided them with the opportunity to process a stimulus presented in a given modality with reference to the material in the other modality. Consequently, this might have resulted in the discrepant results between the pre-tests and the experiment proper.

Interestingly, the fact that the preference for the visual modality, as reflected in GSR findings, was not observed in L2 might indicate that the magnitude of the self-reference effect might be reduced in the non-native language. Such findings might have resulted from a psychological distance when processing L2 compared to L1 (Costa et al. 2014), which was also reflected in the main effect of language, as we found an attenuated skin conductance response to the non-native relative to the native tongue. Such findings are in line with previous research into bilingual emotional language processing, pointing to a decreased sensitivity to L2 emotionally-laden stimuli (Harris 2004; Caldwell-Harris and Ayçiçeği-Dinn 2009; Opitz and Degner 2012; Pavlenko 2012; Costa et al. 2014; Caldwell-Harris 2014; Chen et al. 2015; Hsu et al. 2015). Importantly, such a decreased sensitivity to emotional language in L2 was present even though participants of the current study were proficient in their second language, as confirmed by LexTALE results (Lemhöfer and Broersma 2012). Therefore, the effect found in our experiment might indicate that reduced emotional resonance to L2 may not necessarily be modulated by L2 proficiency level.

It might have however resulted from either late age of L2 acquisition or from a more formal context of L2 acquisition, as our participants acquired their second language around the age of 9 in the formal school setting in Poland. Harris et al. (2006) pointed to both high L2 proficiency level and early AoA as requisite factors in comparable emotional resonance to both native and non-native emotionally-laden stimuli. Therefore, since our participants were sequential bilingual speakers, their L2 language acquisition began once the emotional regulation systems of the brain had already been developed, which might have resulted in a weaker interconnectivity of L2 emotional-laden words with the emotional system of the brain (Bloom and Beckwith 1989; Caldwell-Harris 2014).

Furthermore, the present findings might also provide support for the emotional context of learning hypothesis (Caldwell-Harris 2014), which postulates that emotional responding is modulated by the context of second language acquisition and use, as language acquired via immersion is assumed to be more emotional compared to language learned in a formal school setting (Dewaele 2010; Degner et al. 2012). Future research is however needed in order to further elucidate the role of age of L2 acquisition and the context of L2 acquisition in how bilingual speakers respond to emotionally-laden linguistic stimuli in their native and non-native language.

Importantly, we observed the impact of language and modality on emotional resonance only in galvanic skin responses, and not in the self-report measure (the SUPIN S30 questionnaire). The fact that the analysis of SUPIN scores did not yield similar results might therefore pose a question of reliability of self-reported measures to quantify emotional responding (Mauss and Robinson 2009; Paulhus and John 1998). Although self-reported measures are often employed to test valence, they seem to be more susceptible to bias than markers of emotional arousal, such as the galvanic skin response. Consequently, lack of effect in SUPIN scores may have resulted from the social desirability bias, which is likely to make people unwilling to give true answers to questions related to their current emotional states (Welte and Russell 1993). Namely, as participants were asked to fill in the SUPIN questionnaire as part of the experimental procedures, they were aware that their emotional responding was being tested in the study, and thus they might have self-reported their current emotional state to be affected emotionally by all four emotionally laden stimuli, irrespectively of language nativeness. As a result, we failed to observe any statistically significant differences in self-reported emotional states in reaction to the native and non-native narratives. Furthermore, some items included in the questionnaire, together with their cognitive labels, may have been regarded by participants as sensitive, which resulted in providing invalid reports of the emotions experienced due to the experimental manipulation. Some individuals may have also been unaware of the emotions experienced, which could provide an alternative explanation of the lack of effect of language and modality on emotional reactivity. Therefore, differences between the findings from the GSR and the self-report measures point to the crucial role of research method triangulation, where self-report measures are combined with physiological methods, thus allowing for obtaining more valid data.

The present study was designed to test the role of modality and language nativeness in emotionally-laden language processing by means of employing the galvanic skin response method along with a self-report measure. The GSR findings showed a preference for visual over auditory stimuli, yet only in the native tongue, which could be accounted for by the self-reference effect and its potentially more limited role in the non-native compared to the native language processing. Additionally, we found a more pronounced sensitivity to emotionally-laden texts presented in L1 relative to L2, which can be explained by a psychological distance when processing non-native emotional stimuli due to differences in age as well as context of L2 acquisition. Importantly, the stimuli used in the current study involved texts that were supposed to evoke sadness. Further research is therefore needed in order to examine whether bilingual emotional language processing might be modulated by emotional valence (i.e., positive vs. negative emotions) or emotional arousal within a specific emotion (e.g., fear vs. sadness).
